# Gastrodin Rescues Autistic-Like Phenotypes in Valproic Acid-Induced Animal Model

**DOI:** 10.3389/fneur.2018.01052

**Published:** 2018-12-07

**Authors:** Xiaona Wang, Jing Tao, Yidan Qiao, Shuying Luo, Zhenqin Zhao, Yinbo Gao, Jisheng Guo, Jinghui Kong, Chongfen Chen, Lili Ge, Bo Zhang, Pengbo Guo, Lei Liu, Yinsen Song

**Affiliations:** ^1^Henan Provincial Key Laboratory of Children's Genetics and Metabolic Diseases, Children's Hospital Affiliated to Zhengzhou University, Zhengzhou, China; ^2^Department of Pathology, Children's Hospital Affiliated to Zhengzhou University, Zhengzhou, China; ^3^Center for Translational Medicine, The Sixth People's Hospital of Zhengzhou, Zhengzhou, China

**Keywords:** autism spectrum disorder, gastrodin, mIPSC, **α**5 gABA_A_ receptor, GAT1

## Abstract

Autism spectrum disorder (ASD) is an immensely challenging developmental disorder characterized by impaired social interaction, restricted/repetitive behavior, and anxiety. GABAergic dysfunction has been postulated to underlie these autistic symptoms. Gastrodin is widely used clinically in the treatment of neurological disorders and showed to modulate GABAergic signaling in the animal brain. The present study aimed to determine whether treatment with gastrodin can rescue valproic acid (VPA) induced autistic-like phenotypes, and to determine its possible mechanism of action. Our results showed that administration of gastrodin effectively alleviated the autistic-associated behavioral abnormalities as reflected by an increase in social interaction and decrement in repetitive/stereotyped behavior and anxiety in mice as compared to those in untreated animals. Remarkably, the amelioration in autistic-like phenotypes was accompanied by the restoration of inhibitory synaptic transmission, α5 GABA_A_ receptor, and type 1 GABA transporter (GAT1) expression in the basolateral amygdala (BLA) of VPA-treated mice. These findings indicate that gastrodin may alleviate the autistic symptoms caused by VPA through regulating GABAergic synaptic transmission, suggesting that gastrodin may be a potential therapeutic target in autism.

## Introduction

Autism spectrum disorder (ASD) represents a neurodevelopmental disorder that is characterized by impaired social interaction, repetitive conduct and anxiety-like behaviors (1). Several brain structures and function have been suggested to underlie behavioral abnormalities of ASD including the basolateral amygdala (BLA) (2). We and others have recently demonstrated the remarkable decrease in pyradimal cells and interneurons numbers in the BLA of individuals and animal models of ASD (3, 4). Excitatory/inhibitory synaptic imbalance in the lateral amygdala of valproic acid (VPA)-treated rats plays critical roles in autistic-like features (5). It was shown that decreasing γ-aminobutyric acid (GABA) function within the BLA decreases sociability (6). This evidence indicates that reduced GABAergic activity leads to the manifestation of symptoms associated with ASD (2).

**Graphical Abstract 1:**
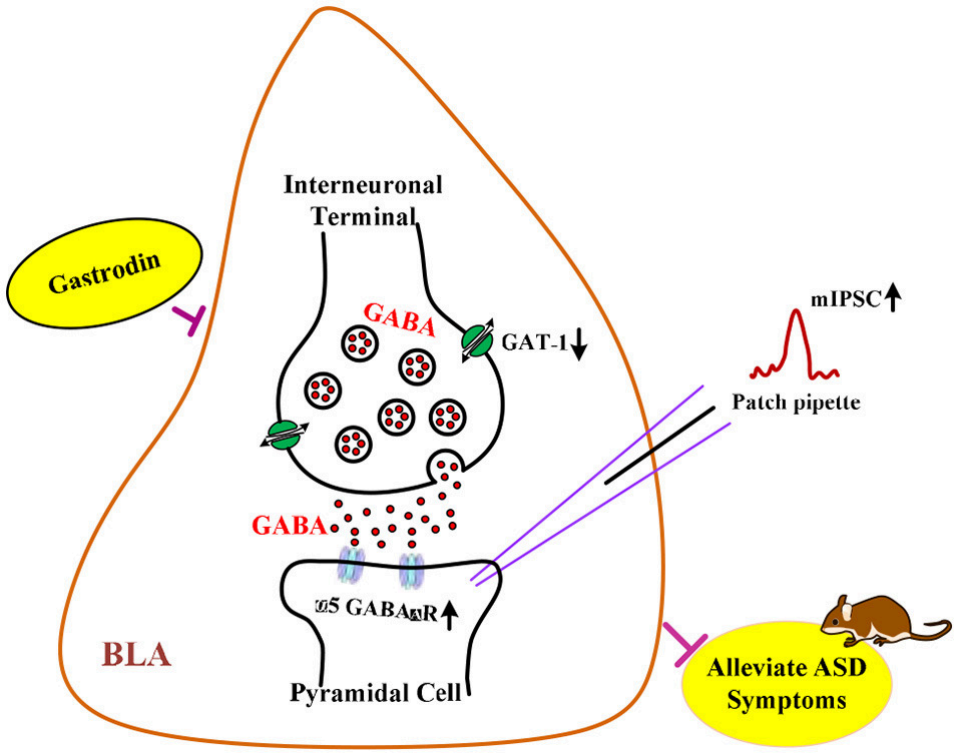
**GRAPHICAL ABSTRACT |** The hypothesis of neurological mechanisms underlying the modulatory effects of gastrodin on ASD-like phenotypes.

GABA is the primary inhibitory neurotransmitter in the adult brain. GABA acting on ionotropic GABA_A_ receptor can regulate neuronal synaptic excitability and social behaviors in ASD (7, 8). Notably, α5 subunit-containing GABA_A_ (α5 GABA_A_) receptor is expressed in vicinity to synaptic GABA release sites of BLA (9). Animal studies have shown that α5 GABA_A_ receptor protein and mRNA expression were decreased in the cortex of ASD (10, 11), which contributes to ASD relevant phenotypes (12, 13). Moreover, emerging research confirms that GABA levels are rapidly degraded by the GABA transporter by which inhibitory neurotransmission and social behaviors are regulated (14–16). Exposure to VPA leads to increased expression of type 1 GABA transporter (GAT1) in the rat amygdala (17). Importantly, numerous studies have reported that drugs alleviate behavioral deficits in animal models of ASD by correcting the excitation/inhibition imbalance in synaptic activity involved in ASD (18, 19).

It has been established that gastrodin has beneficial effects on paralysis, epilepsy, convulsion, and dementia (20). Amounting evidence indicates that gastrodin could possess neuroprotective property, anxiolytic and cognition-enhancing properties. In particular, gastrodin could attenuate anxiety-like behaviors in the rat model of posttraumatic stress disorder (21). Previous data revealed that gastrodin could cause marked elevation of GABA concentrations by inhibiting the GABA shunt in the gerbil hippocampus induced by seizure (22). Studies within our laboratory have demonstrated that prologed gastrodin treatment could rescue 3,3'-iminodipropionitrile-induced cognitive deficits by normalizing GABAergic system (23). Similarly, gastrodin was shown to alleviate impairment in hippocampal synaptic plasticity induced in the lead-treated rats (24). Despite such compelling evidence, it remains uncertain whether gastrodin is involved in the ameliorative effect on ASD-related behavioral abnormalities through GABAergic signaling.

For all these reasons, in the present study, we first investigated whether treatment with gastrodin could prevent autistic-like symptoms induced by prenatal VPA exposure in mice. To explore the underlying mechanisms of relieving autistic symptoms of gastrodin, the regulatory roles of inhibitory synaptic transmission, correlated changes in the α5 GABA_A_ receptor and GAT1 protein expression, were examined within the BLA in VPA-induced animal model.

## Materials and Methods

### Mice

All experiments with animals were approved by the guidelines of the Ethical Committee Experimental Animal Center of Shandong University (animal license No. SCXK20130009) and were performed according to the National Institutes of Health Guide for Care and Use of Laboratory Animals (25). C57BL/6 mice weighing 30–32 g were housed in groups of 3–4 per cage in a temperature-controlled (24°C) animal quarters on a 12:12-h light-dark cycle (lights on at 7:00 a.m.−7:00 p.m). All behavioral observations took place during the light cycle. Mice were mated, with pregnancy designated by the presence of a vaginal plug on embryonic day 1 (E1). Pregnant female mice received a single intraperitoneal injection of sodium salt of valproic acid (NaVPA, 600 mg/kg in 0.9% saline; Sigma-Aldrich, USA) on day E12.5, while control mice received the same volume of saline (19). Animals were housed individually and allowed to remain with their litters until weaning. The male offspring were then separated and housed (3–4 per cage) until the end of all behavioral tests.

### Behavioral Assessment

On postnatal day 21 (P21), gastrodin (100 mg/kg in saline, Sigma) or saline was intragastricaly (i.g.) administered once daily for 15 days based on previous results (26). From day 11 to 15, gastrodin was administered 1/2 h prior to behavioral assays. The behavioral testing was conducted in the saline-treated with vehicle (saline/saline), saline-exposed with gastrodin (saline/GAS), VPA-treated with vehicle (VPA/saline), or VPA-exposed with gastrodin (VPA/GAS) offspring. The social interaction, marble burying, self-grooming, open field and elevated plus maze tests were carried out for five consecutive days after gastrodin treatment. At 24 h after completion of elevated plus maze task, the same offspring received either electrophysiological recordings or were sacrificed for western blot analysis. All behavioral recordings were conducted using ANY-maze software (Stoelting Co. Wood Dale, IL, USA).

### Social Interaction Test

Social interaction between animals was measured as described previously with minor modifications (27). The subject mouse was habituated to the empty social interaction apparatus (30.5 × 24.1 × 21.0 cm) for 5 min. A wired enclosure was then placed in the corner of the social interaction box and an unfamiliar mouse matched by gender and age was placed in the apparatus. The subject mouse was assigned to the social interaction box and allowed to freely interact with the novel mouse for 10 min. The region surrounding the stranger mouse chamber was marked as the area of social interaction (a 5 cm zone surrounding the outside of the wired enclosure). The amount of time spent in the social interaction region, sniffing, and exploring the stranger mouse was recorded and viewed as a measure of social interaction behavior. Average distance traveled in the apparatus measured the total locomotory behavior of mice.

### Marble Burying

Marble burying was performed for testing repetitive and compulsive behavior (28). Empty home cages were filled with 10 cm of bedding, on top of which 12 marbles (1.58 cm in diameter) were evenly spaced in a 3 × 4 grid. The fraction of time spent burying marbles was measured for 15 min. Digging was defined by coordinated movements of fore or hind limbs that displace the substrate. Total number of marbles buried (> 67% marble covered by bedding material) was scored at the end of the testing session.

### Self-Grooming Test

The conditioning chamber consisted of a rectangular box (46 × 23.5 × 20 cm) with 2 cm of bedding. The time mice spent grooming was measured for 10 min. Grooming behavior was considered as scratching or stroking of the face, head, or body with the two forelimbs or licking body parts (18).

### Open Field Test

The open field test was performed as previously described (27). Each mouse was placed in the corner of the open field apparatus (50 × 50 × 40 cm) facing the wall. The subject was given a 10 min test. The time spent in the center (15 × 15 cm imaginary square) and total distances traveled were measured for 10 min.

### Elevated Plus Maze

The elevated plus maze measures anxiety-like behavior in rodents (29). The mice were placed in a standard elevated plus maze for 15 min, which was composed of a plus-shaped apparatus with two opposite open arms and two enclosed arms (60 × 5 × 30 cm) arranged at right angles. The percentage of time spent in each arm and the total distances (cm) moved were measured.

### Electrophysiology

The slices (400 μm) of the BLA were prepared from male mice using a Vibroslice (Leica VT 1,000 S) in an ice-cold solution (mM) that contained 234 sucrose, 2.5 KCl, 0.1 CaCl_2_, 4 MgSO_4_, 1 NaH_2_PO_4_, 15 Hepes and 11 glucose. Slices were allowed to equilibrate for at least 1 h at room temperature (22–24°C) in an artificial cerebro-spinal fluid (ACSF) solution (mM) containing 130 NaCl, 26 NaHCO_3_, 3.0 KCl, 1.25 NaH_2_PO_4_, 1.0 CaCl_2_, and 10 glucose. A single slice was then transferred to a submerged recording chamber and perfused with ACSF (3–4 ml/min). All solutions were saturated with 95% O_2_/5% CO_2_.

BLA pyramidal cells were visualized with an infrared-sensitive CCD camera with a 40 × water-immersion lens (Zeiss, Axioskop2 FS Plus) and recorded using whole-cell techniques (MultiClamp 700B Amplifier, Digidata 1320 A analog-to-digital converter) and pClamp 10.2 software (Molecular Devices). Miniature inhibitory postsynaptic current (mIPSC) were recorded in the presence of 30 μm D (-)-2-Amino-5- phosphonopentanoic-acid (AP5; Sigma), 20 μm 6- cyano-7-nitroquinoxaline-2,3-dione (CNQX; Tocris Bioscience, UK) and 1 μm tetrodoxin (TTX, Sigma). To record mIPSC, pipettes were filled with the following solution (mM): 140 CsCl, 4 MgATP, 3 NaGTP, 10 Hepes, 1 EGTA, 1 MgCl_2_, 0.3 CaCl_2_ (pH 7.25, 285 mOsm). The resistance of the pipettes was 3–6 MΩ. The holding potential for mIPSC was −70 mv. Data were collected when series resistance fluctuations remained within 15% of the initial value (10–15 MΩ). Signals were sampled at 10 kHz and filtered at 2 kHz.

### Western Blotting Analysis

BLA samples were homogenized in 150 μl of homogenizing buffer containing 50 mm Tris (pH 7.4), 150 mm NaCl, 1 % Triton X-100, 1% sodium deoxycholate, 0.1 % SDS, 1 mm Na_3_VO_4_, 1 mm EDTA, and 1 mm PMSF. The supernatant was collected and protein content was determined with the BCA Protein Assay Kit (Cowin Biotech, Beijing, China). Total protein (30–50 μg) was separated by SDS-PAGE and transferred to PVDF membrane. The membranes were incubated with 5% non-fat dried milk for 60 min and then incubated in the primary antibodies of α5 GABA_A_ receptor (1:1000; Abcam, Cambridge, UK), GAT1 (1:2000; Abcam, Cambridge, UK) and GAPDH (1:1000; Millipore, Milford, MA, USA) overnight at 4°C. The membranes were visualized with horseradish peroxidase-conjugated goat anti-rabbit/mouse IgG (1:5000; ZSGB-Bio, Beijing, China) for 2 h at room temperature. Membranes were developed using the ECL detection Kit (Thermo Fisher Scientific, Rockford, IL, USA). BLA samples from six mice were repeated in triplicate. The protein bands were scanned and quantified using ImageJ software.

### Statistical Analysis

Data were analyzed by two-way ANOVA followed by the Tukey's test when appropriate. Data are expressed as the means ± SEM. Statistical analyses were carried out in GraphPad Prism 7.03. The level of statistical significance was set at *p* < 0.05.

## Results

### Gastrodin Mitigates Social Interaction Deficit of VPA-Exposed Mice

**Figure 1:**
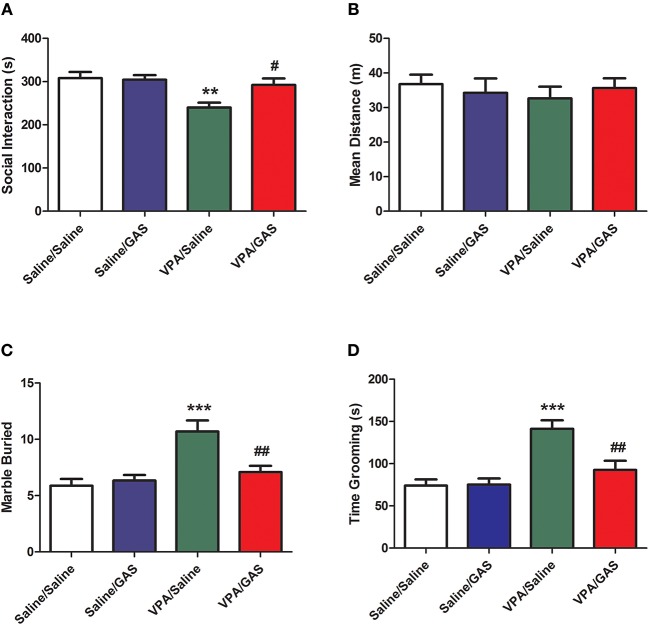
Amelioration of social interaction deficit and repetitive/stereotyped in VPA-exposed offspring by gastrodin. On P31-33, mice were administered the social interaction, marble burying and grooming tests. **(A, B)** In the social interaction test, the duration of social interaction and average distance were measured in the VPA- and saline-treated offspring for 10 min. **(C)** In the marble burying test, the number of marbles buried was measured for 15 min. **(D)** In the grooming test, the time spent grooming was measured for 10 min. Values represent means ± SEM (*n* = 8). ^***^p < 0.001, ^**^p < 0.01 vs. Saline/Saline. ^##^*p* < 0.01, ^#^*p* < 0.05 vs. VPA/Saline.

The mice were divided into saline/saline, saline/GAS, VPA/saline, and VPA/GAS 4 groups. We determined whether gastrodin had any effect on social interaction deficit in VPA-treated offspring. We performed behavioral test to assess social interaction deficit, a prominent symptom of ASD (10). As shown in Figure 1A, ANOVA revealed the effect of VPA [*F*_(1, 28)_ = 6.452, *p* < 0.05], GAS [*F*_(1, 28)_ = 6.452, *p* < 0.05], and the VPA × GAS interaction [*F*_(1, 28)_ = 8.046, *p* < 0.05]. The offspring exposed to VPA exhibited the lower duration of social interaction than saline-treated mice (*p* < 0.01). In contrast, the impairment in social interaction was completely reversed after treatment with gastrodin (*p* < 0.05). However, there were no differences in mean distance of social interaction behavior between the groups [*F*_(3, 28)_ = 0.838, *p* > 0.05, Figure 1B]. These data indicate that prenatal exposure toVPA triggers social disturbance in ASD and gastrodin treatment can overcome it.

### Gastodin Alleviates Repetitive and Stereotyped Behavior in Autistic Mice

To identify the therapeutic effect of gastrodin in the stereotyped behavior of VPA-exposed mice, we next calculated the total number of marbles buried of marble-burying behavior. As shown in Figure 1C, ANOVA revealed the effect of VPA [*F*_(1, 28)_ = 16.544, *p* < 0.001], GAS [*F*_(1, 28)_ = 5.263, *p* < 0.05], and the VPA × GAS interaction [*F*_(1, 28)_ = 8.716, *p* < 0.01] on the total number of marbles buried. Mice subjected to VPA showed increased the total number of marbles buried when compared to control mice (*p* < 0.001), whereas these increases were reverted by repeated treatment with gastrodin (*p* < 0.01).

We further investigated the ameliorative effect of gastrodin in the repetitive self-grooming behavior which is other core symptom of ASD (30). ANOVA revealed the effect of VPA [*F*_(1, 28)_ = 21.834, *p* < 0.001], GAS [*F*_(1, 28)_ = 6.899, *p* < 0.05], and the VPA × GAS interaction [*F*_(1, 28)_ = 7.658, *p* < 0.01] on the time spent grooming (Figure 1D). The results showed that VPA exposure significantly increased the time spent grooming compared with the corresponding controls (*p* < 0.001). Importantly, gastrodin treatment restored the excessive self-grooming to the normal level (*p* < 0.01). Altogether, these results show that VPA may also produce the repetitive/stereotyped behavior, and gastrodin treatment can suppress the marble burying and excessive self-grooming behavior.

### Gastrodin Reverses Anxiety-Related Behavior in VPA-Treated Mice

**Figure 2:**
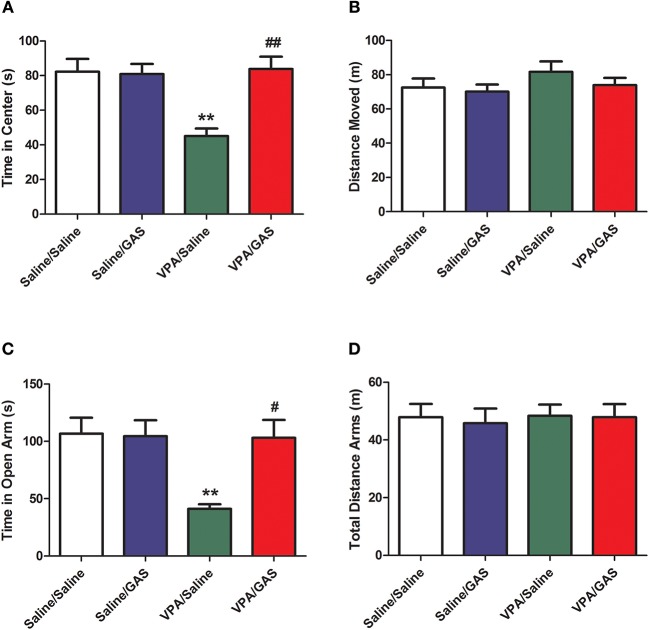
Reversal of anxiety-like behavior in VPA-exposed offspring by gastrodin. On P34-35, mice were administered the open field and elevated plus maze tests. **(A, B)** In the open field test, the time spent in the center and distance traveled were measured for 10 min. **(C, D)** In the elevated plus maze test, the time spent in the open arms and distance traveled were measured for 15 min. All data represent means ± SEM from 8 mice per group. ^**^p < 0.01 vs. Saline/Saline. ^##^*p* < 0.01, ^#^*p* < 0.05 vs. VPA/Saline.

ASD was shown to display anxiety-related behavior (31). Next, we tested the effect of a systemic injection of gastrodin on two paradigms commonly used to determine anxiety-like behavior: open field and elevated plus maze (32). As illustrated in Figure 2A, in the open field, ANOVA revealed the effect of VPA [*F*_(1, 28)_ = 4.352, *p* < 0.05], GAS [*F*_(1, 28)_ = 13.271, *p* < 0.01], and the VPA × GAS interaction [*F*_(1, 28)_ = 14.160, *p* < 0.01] on the time spent in the center. Compared to saline-injected mice, VPA-exposed mice decreased the time spent in the center of open field (*p* < 0.01). Interestingly, the decrease of time spent in the center was attenuated by treatment with gastrodin (*p* < 0.01). Whereas, no significant differences in distance moved were detected between groups [*F*_(3, 28)_ = 0.928, *p* > 0.05, Figure 2B].

Additionally, in the elevated plus maze, ANOVA revealed the effect of VPA [*F*_(1, 28)_ = 6.566, *p* < 0.05], GAS [*F*_(1, 28)_ = 5.430, *p* < 0.05], and the VPA × GAS interaction [*F*_(1, 28)_ = 6.032, *p* < 0.05] on time in open arm (Figure 2C). Our results showed that VPA exposure markedly decreased the time in open arm (*p* < 0.01), treatment with gastrodin significantly ameliorated such reduction of time in open arm as compared to the VPA-treated mice (*p* < 0.05). Nevertheless, no significant differences in total distance arms were observed between groups [*F*_(3, 28)_ = 0.135, *p* > 0.05, Figure 2D]. Accordingly, these data support the idea that gastrodin treatment can attenuate anxiety-like abnormality induced by VPA.

### Gastrodin Prevents VPA-Induced Inhibitory Synaptic Impairment

**Figure 3:**
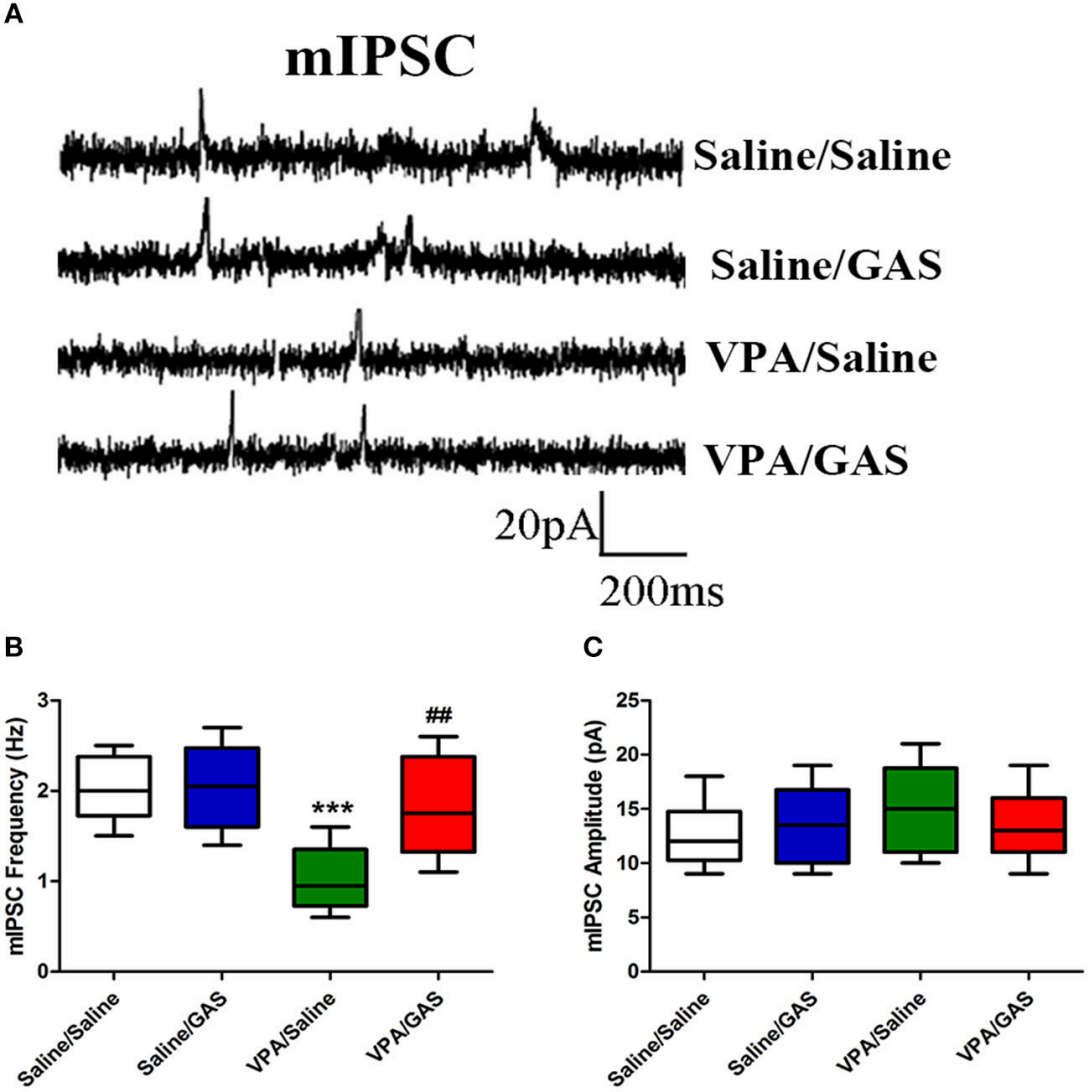
Effect of gastrodin on the frequency and amplitude of mIPSC recorded in the BLA of VPA-treated mice. **(A)** Sample traces of mIPSC taken from slices of Saline/Saline, Saline/GAS, VPA/Saline, and VPA/GAS mice. mIPSC were recorded in the BLA pyramidal cells at a holding potential of −70 mV in the presence of TTX (1 μm). **(B, C)** Summary plots of the frequency and amplitude of mIPSC in the Saline/Saline, Saline/GAS, VPA/Saline, and VPA/GAS mice. All data represent means ± SEM from 8 mice per group. ^***^p < 0.001 vs. Saline/Saline. ^##^*p* < 0.01 vs. VPA/Saline.

A decrease in synaptic inhibition has been demonstrated in animal models of ASD, which results in the excitation/inhibition imbalance (33). To explore the mechanisms underlying the protective effects of gastrodin against VPA induced autistic-like behavioral phenotypes, we further examined whether altered GABAergic synaptic transmission in VPA-treated offspring could be normalized by gastrodin. Whole-cell recordings were made from the soma of visually identified pyramidal-like neurons located in the BLA. As shown in Figures 3A–C, ANOVA revealed the effect of VPA [*F*_(1, 28)_ = 15.797, *p* < 0.001], GAS [*F*_(1, 28)_ = 6.397, *p* < 0.05], and the VPA × GAS interaction [*F*_(1, 28)_ = 5.997, *p* < 0.05] on mIPSC frequency. Figure 3B shows that VPA/Saline mice exhibited significantly the lower frequency of mIPSC than saline/saline groups (*p* < 0.001). As expected, administration of gastrodin effectively ameliorated mIPSC frequency of VPA-treated mice to the similar extent as those of the control subjects (*p* < 0.01). The amplitude of mIPSC did not differ among the four groups [*F*_(3, 28)_ = 0.624, *p* > 0.05] (Figure 3C). The results suggest that VPA decreases inhibitory synaptic transmission. Gastrodin treatment reinstates inhibitory synaptic transmission to the physiological level.

### Alterations of α5 GABA_A_ Receptor and GAT1 Levels After Gastrodin Treatment

**Figure 4:**
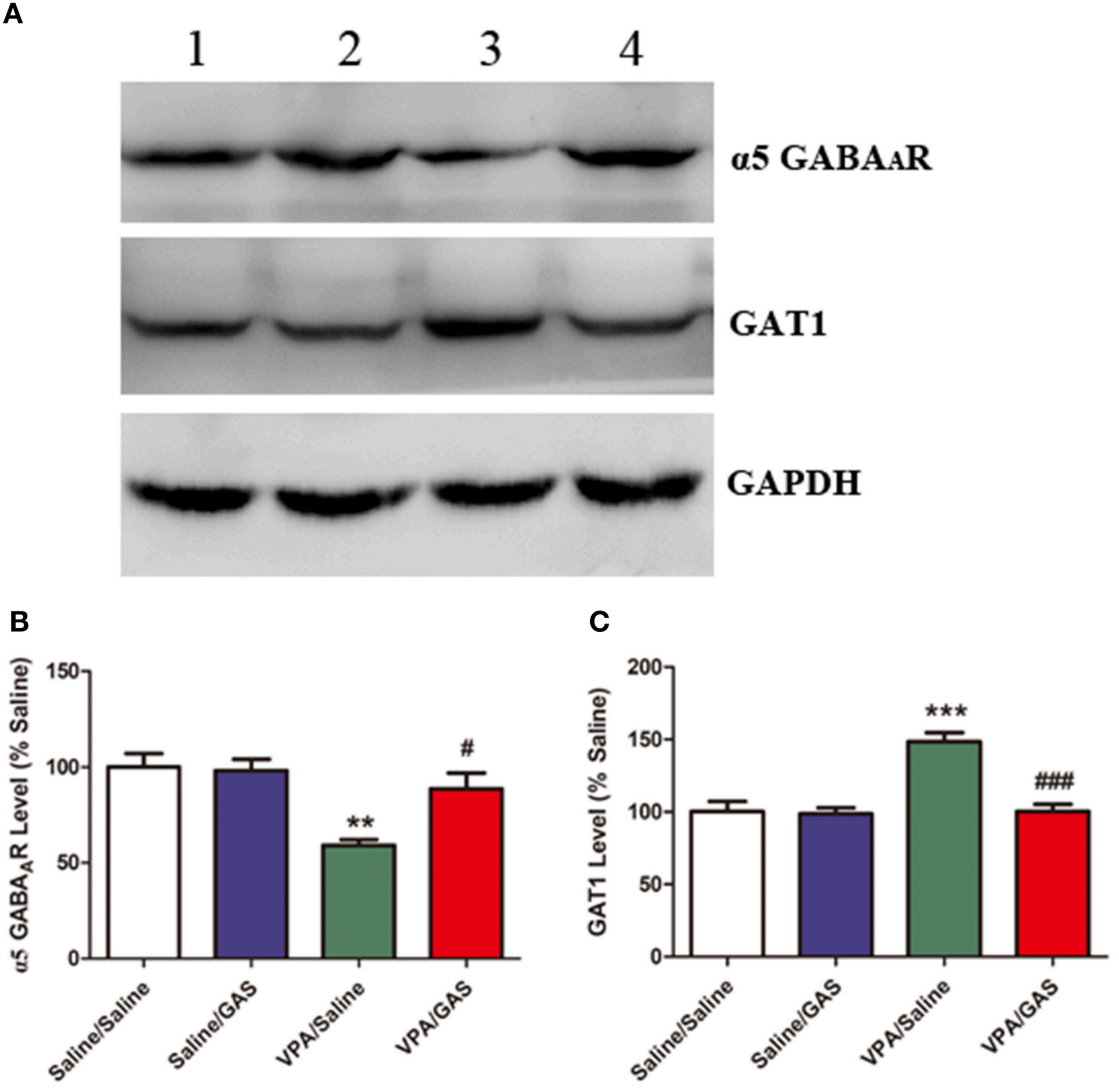
Effect of gastrodin on expression of α5 GABA_A_ receptor and GAT1 protein in the BLA of VPA-treated mice. **(A, B)** Band 1: Saline/Saline; Band 2: Saline/GAS; Band 3: VPA/Saline; Band 4: VPA/GAS. **(C)** Normalized intensity bands of α5 GABA_A_ receptor and GAT1 are shown as means ± SEM of at least six separate experiments. Results indicated the percentage changes from saline-exposed group. ^*^p < 0.01 vs. saline-exposed group. ^***^p < 0.001 vs. saline-exposed group; ^##^*p* < 0.01, ^###^*p* < 0.001 vs. VPA- treated group.

Based on the aforementioned electrophysiological experiments which showed that the mIPSC frequency decreased in mice subjected to VPA, we examined whether this change was due to changes in GABAergic system, including α5 GABA_A_ receptor and GAT1. ANOVA revealed the effect of VPA [*F*_(1, 28)_ = 19.416, *p* < 0.001], GAS [*F*_(1, 28)_ = 5.462, *p* < 0.05], and the VPA × GAS interaction [*F*_(1, 28)_ = 6.691, *p* < 0.05] on α5 GABA_A_ receptor protein expression. The results showed that prenatal VPA exposure significantly decreased α5 GABA_A_ receptor expression in the BLA compared with the corresponding controls (*p* < 0.001). However, administration of gastrodin effectively reversed VPA-induced the reduction of α5 GABA_A_ receptor expression (*p* < 0.01, Figures 4A,B).

Further, as shown in Figures 4A,C, ANOVA revealed the effect of VPA [*F* (1, 28) = 20.189, *p* < 0.001], GAS [*F*_(1, 28)_ = 19.788, *p* < 0.001], and the VPA × GAS interaction [*F*_(1, 28)_ = 17.281, *p* < 0.001] on GAT1 protein expression. In specific, *post-hoc* analysis indicated that GAT1 expression of VPA-treated mice in the BLA was significantly increased as compared to saline groups (*p* < 0.001). However, gastrodin treatment markedly normalized the alteration in GAT1 expression as compared with VPA alone (*p* < 0.001). These results that prenatal VPA exposure altered α5 GABA_A_ receptor and GAT1 expression, which may contribute to the decreased inhibitory neurotransmission. While gastrodin reverses the abnormal expression of protein back to the normal level.

## Discussion

Our study confirm that offspring exposed VPA display autistic-like phenotypes and demonstrate that these behavioral manifestations are associated with the abnormal decrease in inhibitory neurotransmission and abnormal expression of synaptic α5 GABA_A_ receptor and GAT1. Most importantly, we present results that gastrodin treatment for 14 days effectively reverses the decreased GABAergic transmission and abnormal expression of α5 GABA_A_ receptor and GAT1, as well as overcomes autistic-like behaviors caused by VPA. Indeed, no evidence of neuronal degeneration was seen in the H&E-stained sections in the gastrodin-treated mice (Figure S1). We have therefore provided evidence that gastrodin may restore the GABAergic transmission via modulating synaptic receptor and transporter and subsequently ameliorating ASD relevant phenotypes.

In this study, gastrodin was capable of ameliorating VPA induced autistic behaviors in the social interaction, marble burying, self-grooming, open field, and elevated plus-maze tests, which are associated with social interaction, repetitive/stereotyped behavior and anxiety involved neuronal circuits in the BLA (2). Consistent with the prior research (31, 34), our data showed that prenatal exposure to VPA induced ASD-like behaviors in mice. More importantly, we found that gastrodin treatment potently reversed such deficits as shown by the prolonged time spent in the center, shortened time in spent burying and grooming in VPA-exposed mice, indicative of the beneficial effects of gastrodin on the autistic relevant defects. Similarly, Peng et al. (21) repeated that treatment with gastrodin produced the anxiolytic effect in rat model of posttraumatic stress disorder. Take together, the present study further proposed that gastrodin exhibited the significant amelioration of behavioral deficits in VPA-exposed offspring.

GABAergic dysfunction has been postulated to underlie the manifestation of symptoms associated with ASD in the BLA (18, 35). Animal studies showed that GABAergic neurotransmission was severely compromised in rodents prenatally exposed to one high dose of VPA (600mg/kg) (36). Virus-mediated deletion of inhibitory synapse-specific neuroligin-2 in the prefrontal cortex results in decreases in inhibitory synapse density and mIPSC frequency, effects that are accompanied by social deficit (37). Noticeably, GABA dysfunction or alterations in excitatory/inhibitory balance have been implicated in ASD-relevant behaviors may be treated by drugs that potentiate GABAergic transmission (38, 39). Roberto et al. (19) suggested that cerebrolysin treatment can reverse the behavioral deficits and prevent the impairment of inhibitory synapses of VPA offspring. In addition, Chen et al. (20) showed that the protective role of gastrodin on abnormal hyperexcitability may be partially mediated by its inhibiton on Aβ_1−42_-elicited inward currents in entorhinal cortex neurons. It was proposed that gastrodin can reverse the pathologically altered Na^+^ and K^+^ currents in small dorsal root ganglion neurons in rat model of diabetes (40). Of particular noteworthy, we found gastrodin treatment reversed the decreased mIPSC frequency in VPA-exposed offspring, accompanied by the alleviation of behavioral abnormalities. Accordingly, our data strongly suggest that gastrodin treatment may ameliorate ASD-relevant phenotypes induced by VPA though enhancement of GABAergic neurotransmission. Additionally, we have previously shown that gastrodin treatment attenuated 3,3′ -iminodipropionitrile-induced cognitive impairments, which suggest the involvement of dopaminergic and serotoninergic system (23). It is further proposed that ameliorative effects of gastrodin on VPA -induced behavioral deficits in autism could be related to dopaminergic and serotoninergic alterations as well.

Several lines of evidence suggests that reduction in the α5 GABA_A_ receptor expression and activity has been implicated in the etiology of ASD (13). Previous work involving positron emission tomography (PET) has demonstrated the reduced binding of α5 GABA_A_ receptor-selective ligand in amygdala of autistic subjects (41). Genetically modified mice that lack α5 GABA_A_ receptor mice exhibit the reduced tonic inhibitory conductance and enhanced excitability of principal cells, contribute to behavioral phenotypes of ASD (12, 42, 43). Moreover, α5 GABA_A_ receptor appears to preferentially modulate non-somatic compartments of BLA pyramidal cells and thus modulate synaptic excitation/inhibition balance and timing established by feedforward inhibition (38, 44). Indeed, Han et al. (33) demonstrated that incrasesing GABA tone with low doses of clonazepam benzodiazepine (GABA_A_ receptor agonist) improves characteristic ASD-like behaviors in the BTBR T^+^Itpr3^tf^/J model mice of ASD. Most prominently, we observed that α5 GABA_A_ receptor expression in VPA-exposed offspring was substantially down-regulated, which may lead to suppression of GABAergic transmission. Furthermore, treatment with gastrodin impeded the decrease in α5 GABA_A_ receptor expression following VPA exposure. Therefore, our findings confirm that alteration in α5 GABA_A_ receptor expression might be responsible for the alleviation of autistic symptoms by gastrodin in VPA-treated offspring.

Pharmacological and genetic manipulations of GAT1 could modify anxiety and GABAergic synaptic transmission in rodents (15, 17). In particular, previous studies demonstrated that GAT1 knockout (GAT1^−/−^) mice have phenotypes of hyperactivity and lower anxiety (45). Similarly, Günther et al. (46) reported that GAT1 mRNA expression shows a transient increase followed by a decrease after kainic acid-induced seizures. It was shown that tonic GABA_A_ receptor-mediated current is substantially increased in the hippocampus of GAT1^−/−^ mice (15). Blockade of GAT1 also increases the duration of evoked IPSC at inhibitory synapses (47). In aggrement with this notion, we found that administration of VPA induced impairment of inhibitory synaptic transmission and ASD-related phenotypes, accompanied by up-regulated GAT1 expression in the BLA. It is worth noting that we found that gastrodin treatment suppressed the increase of GAT1 expression caused by VPA. Collectively, these data strongly suggest that gastrodin treatment may protect mice from VPA induced ASD-related defects though regulating GAT1 expression.

We conclude that prolonged treatment of gastrodin can ameliorate VPA-induced autistic phenotypes by enhancement of inhibitory neurotransmission, accompanied with alterations in α5 GABA_A_ receptor and GAT1 expression. This indicates that gastrodin may be used as a potential novel targeted therapeutic strategy for ASD.

## Availability of Data and Materials

The datasets used and/or analyzed during the current study are available from the corresponding author on reasonable request.

## Ethics Statement

All mouse experimental protocols were followed according to NIH guidelines and approval from the Animal Care and Use Committee of the Shandong University.

## Author Contributions

XW contributed to the study design, analyzed and interpreted the data, compiled all the figures, and wrote the manuscript. JT and YQ assisted with the preparation of the ASD model mice and participated in the preparation of the manuscript. CC, LG, BZ, PG, and LL performed behavioral characterization and analyzed the data. YG conducted electrophysiological experiments in slice and analyzed the data. JK and YS carried out and analyzed the Western blot experiments. JG assisted with the statistical analysis, contributed to the data interpretation, and critically revised the manuscript. SL and ZZ participated in the design and coordination of the study and critically revised the manuscript. All authors have read and approved the final version of the manuscript.

### Conflict of Interest Statement

The authors declare that the research was conducted in the absence of any commercial or financial relationships that could be construed as a potential conflict of interest.
