# Supplementary material for: Gastrodin Rescues Autistic-Like Phenotypes in Valproic Acid-Induced Animal Model
Source: Front Neurol. 2018 Dec 7;9:1052. doi: 10.3389/fneur.2018.01052 (PMC6293267; doi:10.3389/fneur.2018.01052)
Supplement: Supplementary file 1 [file Data_Sheet_1.doc]

**Supplemental Information**

**Supplemental Experimental Procedures**

**Hematoxylin-eosin (HE) staining**

BLA tissues were immersed in ice-cold 4% phosphate-buffered paraformaldehyde for 24 h, transferred to 70% ethanol, dehydrated at 70, 90, 95 and 100% for 2 min, embedded in paraffin wax blocks, and serially sectioned into 4 µm-thick segments. Tissue sections were dewaxed in xylene, rehydrated through decreasing concentrations of ethanol. The sections were then stained with 0.5% hematoxylin for 5 min, followed by 0.5% eosin for 30 sec at room temperature. Stained cells were observed using a light microscope (Leica DM500 ICC50).

**
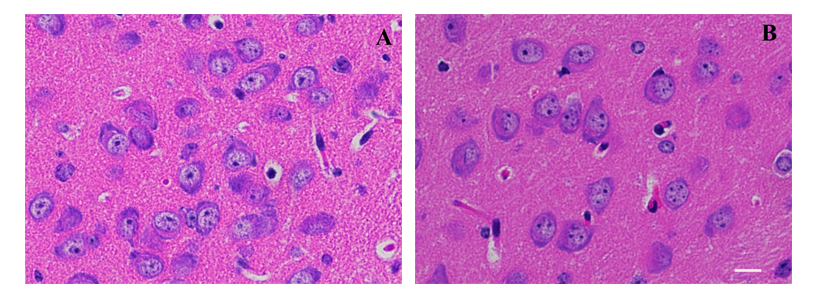
**

**Figure legend**

Fig.S1. H&E-stained neurons region from P21 offsprings administration of saline (A) and gastrodin (B) for 15 days in the BLA. Scale bars, 20 μm.
